# Does Lack of Vertical Transmission of COVID-19 Guarantee the Health of the Fetus or Neonate in Infected Mothers?

**DOI:** 10.18502/jri.v21i4.4323

**Published:** 2020

**Authors:** Mohammad Reza Sadeghi

Although in SARS-CoV-2 infection, the aged people as a high-risk group are exposed to respiratory and related systemic diseases, pregnant women should also be regarded as a high-risk population. This issue should be placed at the core of public health strategies focusing on prevention and treatment of SARS-CoV-2 infection.

In COVID-19 outbreak, infected pregnant women appear to have fewer maternal and neonatal complications than those pregnant women infected with other respiratory viruses, such as H1N1 influenza and SARS-CoV and MERS-CoV. Infection of pregnant women with H1N1, SARS and MERS has been reported to be the cause of severe dysfunction of some organs and eventually death of mother and fetus. Having COVID-19 infection during the first or second trimester can lead to miscarriage, premature birth, birth defects and other congenital infections (1).

Pregnant women need more attention due to physiological changes and their susceptibility to disease during the outbreak of COVID-19. Changes in the immune system, increased metabolism and oxygen consumption, cardio-vascular changes and increased ACE2 expression during pregnancy are physiological causes making pregnant women susceptible to viral diseases (2).

The placenta is a complex and unique organ with a critical function in proper growth and development of the fetus during pregnancy. The placenta acts like a heart, lung, liver and kidney for the fetus, and is a part of the in-nate immune system; its main role is to prevent the transmission of pathogens from mother to fetus. Syncytio-trophoblast cells, viz placental epithelial cells, act as a physical barrier against viruses and other infectious agents. SARS-CoV-2 infection causes inflammatory and vascular changes in the placenta including decidual arterio-pathy, fibrinoid necrosis, and amniotic membrane arteriole hypertrophy (3).

Altogether, the outcomes on fetus and neonates of infected mothers are largely unclear. However, SARS-CoV-2 is unlikely to be transmitted across the placenta but vertical transmission of some pathogens from mother to fetus can lead to serious complications and damage. Vertical infection can occur prenatal, per-partum or postnatal. Prenatal transfer has different consequences depending on the gestational age. The severity of fetal injuries is very high in the first trimester, while sometimes maternal infections in the second and third trimesters manifest with immunological symptoms or preterm labor. SARSCoV-2 is a highly pro-inflammatory infection that may cause similar changes in neonatal inflammation and immunity. The presence of IgM in fetus or umbilical cord is indicative of vertical transmission and subsequent intrauterine infection. Nevertheless, detection methods for IgM are prone to inaccuracies due to cross-reactivity with rheumatoid factors or non-specific IgM antibodies (4).

There is always the possibility of the indirect negative effects of COVID-19 on the fetus of the infected mother through the maternal immune system. Maternal infection produces a broad immune response that is related to an inflammatory response associated with excessive secretion of cytokines (Cytokine storms) and severe activation of cellular immunity. Cytokine storm increases the levels of humoral proinflammatory compounds such as inter-leukin-6 (IL-6), interferon gamma (IFN-Y), MCP-1/CCL2, IL-1, IL-12, IL-8, TNFα, and CXCL 10 activates the maternal immune system and increases the pathogenesis of COVID-19. In addition, some of these cytokines can cross the placental barrier and stimulate inflammatory responses in the fetus, which may lead to damage of multiorgan system with negative effects on fetal development (5).

According to one study, pregnant women with SARS-CoV-2 infection have higher levels of IL-6 than non-pregnant women; this increase in IL-6 levels subsequently activates a cascade of proinflammatory reactions that reduces synthesis of growth hormone and IGF-1 in the placenta. IGF-1 deficiency during uterine growth or after childbirth can cause autistic brain damage in the neonate. However, it is suggested that the role of IL-6 is determining in the pathogenesis of neurodevelopmental disorders. IL-6 has been considered as an indicator of maternal systemic inflammation with the potential to affect placental and fetal interactions, subsequent fetal brain development, and risk of psychiatric disorders. Therefore, it seems that maternal inflammation provides intra-uterine conditions that are associated with potential psychiatric and neurological disorders (6).

SARS-CoV-2 may indirectly lead to long term side effects on fetal neurodevelopment by affecting maternal immune activation (MIA). MIA and inflammation show a wide range of short-term and long-term adverse outcomes in neonates. In addition, the association between maternal immune activation-induced autism and schizophrenia has been well established through epidemiological studies and animal models (3).

Therefore, the effect of this virus on people in different age, sex and ethnic groups and even on different organs of an infected person is unknown and every day new symptoms and complications of this disease are reported. Due to the lack of knowledge about its effects on the fetus, neonates, and infected mothers, pregnancy should be delayed as much as possible. At the same time, by conducting more studies, our knowledge can be consolidated regarding the short and long-term effects of this virus on both mother and fetus during pregnancy. Currently, published studies with small sample size are often performed on infected women at late phases of pregnancy (Third trimester). Therefore, special attention should be paid to early stages of pregnancy, during which the virus affects the placental functions with subsequent risks for the fetus. In addition, further research on the inflam-matory disorders in pregnant women with SARS-CoV-2 and longitudinal studies on newborns, exposed to the virus directly or indirectly, are required to ensure proper care of infected pregnant women and their newborns.

It seems the coronavirus will be with us for many years to come, and we need to know more about living with this unknown virus with maximum protection and suppress its harmful effects.
